# Ningdong Granule Upregulates the Striatal DA Transporter and Attenuates Stereotyped Behavior of Tourette Syndrome in Rats

**DOI:** 10.1155/2020/2980705

**Published:** 2020-09-12

**Authors:** Jijun Li, Yi Guo, Lin Zhao, Kexing Sun, Guangjun Xi, Zai-Wang Li

**Affiliations:** ^1^Department of Integrative Medicine on Pediatrics, Shanghai Children's Medical Center, Shanghai Jiao Tong University School of Medicine, Shanghai 200127, China; ^2^Department of Neurology, Shenzhen People's Hospital, The Second Clinical Medical College of Jinan University, The First Affiiated Hospital of Southern University of Science and Technology, Shenzhen 518020, China; ^3^Department of Integrative Medicine on Pediatrics, Provincial Hospital Affiliated to Shandong University, Jinan, Shandong 250021, China; ^4^Department of Neurology, Wuxi People's Hospital of Nanjing Medical University, Wuxi 214023, China

## Abstract

This study aimed to evaluate the possible mechanism of Ningdong granule (NDG) for the treatment of Tourette syndrome (TS). The rats with stereotyped behavior were established by microinjection with TS patients' sera; then, the model rats were divided into NDG and haloperidol (Hal) group, and the nonmedication model rats were regarded as treatment control (TS group). The stereotyped behavior of the rats was recorded, the level of dopamine (DA) in striatum, and the content of homovanillic acid (HVA) in sera were tested, and dopamine transporter (DAT) expression was measured in the study. The experimental results showed that NDG effectively inhibited the stereotyped behavior (*P* < 0.01), decreased the levels of DA in the striatum (*P* < 0.05), increased the content of sera HVA (*P* < 0.01), and enhanced the protein and mRNA expression of DAT in the striatum (*P* < 0.01). Additionally, the results also revealed Hal could improve the stereotyped behavior as well but had no remarkable influence on DAT expression and DA metabolism. In conclusion, NDG attenuates stereotyped behavior, and its mechanism of action might be associated with the upregulation of DAT expression to regulate DA metabolism in the brain.

## 1. Introduction

TS is a chronic neurobehavioral and neuropsychiatric disorder that is characterized by multiple, stereotyped, involuntary, purposeless, and repetitive movements and vocal tics with a male gender bias, which waxes and wanes spontaneously for years [1–6]. There are approximately four to 10 per 1000 school-aged children and adolescents suffering from TS [2, 4, 7, 8]. Attention deficit hyperactivity disorder (ADHD) and obsessive compulsive disorder (OCD) are usually associated with TS as common comorbidities [2, 4, 9, 10].

The full pathophysiological mechanism of TS is not well understood. However, functional neuroimaging and neurobiological studies have provided evidence for the involvement of a metabolic and transmission disturbance of DA within the cortico-striatal-thalamo-cortical (CSTC) circuits [11–13]. Furthermore, the presence of autoantibodies reacting in the brain is also thought to be mechanistically involved in the pathophysiology of TS. The presence of these autoantibodies strongly favors the autoimmune and immune dysregulation hypothesis of TS [14–17]. Haloperidol (Hal), a dopamine D_2_ receptor (DRD_2_) blocker, is effective at improving tic symptoms [18]. Nevertheless, a considerable number of TS children cannot tolerate the adverse side effects of Hal [19, 20]. This difficulty calls for an alternative, safe, and effective medication for TS children. As a traditional Chinese medicine (TCM) preparation, Ningdong granule (NDG) can reduce stereotyped behavior and regulate metabolic DA in TS animals and ADHD patients [5, 21–24], but its full mechanism remains obscure.

Several studies demonstrated that hyperfunction of the dopamine system plays an important role in the pathogenesis of TS, and dopamine transporter (DAT) is the key transporter regulating the function of dopamine system [4, 5]. NDG can effectively alleviate the clinical symptoms of the children with TS [22–24]; therefore, we hypothesized that NDG might modulate DAT expression to regulate DA metabolism in the brain and, as a result, to attenuate stereotyped behavior in TS model rats in the study.

## 2. Animals and Methods

### 2.1. Ethics Statement

Written consent was obtained from children's guardians to collect TS sera for use in this study. The whole research procedures were deliberated and approved by the Medical Ethics Committee of Shanghai Children's Medical Center, Shanghai Jiao Tong University School of Medicine. The animal study was also approved by the ethics committee of experimental animal of committee of Shanghai Children's Medical Center, Shanghai Jiao Tong University School of Medicine.

### 2.2. Experimental Animals

Forty-eight male Wistar rats (6-week-old, 160 ± 20 g) were purchased from the Shandong Experimental Animal Center (Jinan, China) and were housed in an air-conditioned animal room with a 12-hour light/dark cycle, a temperature of 22 ± 2°C, and a humidity of 50 ± 10%. The rats were provided with free access to food and tap water and were maintained for one week before starting the experiment. Previous studies have also suggested that there is an increase in stereotyped behavior in rats after the infusion of TS sera rich in antineural antibodies into the ventrolateral striatum [26–28]. Thirty-six stereotyped behaviors were induced in the rats as the models by microinfusing TS patients' sera containing antineural antibodies according to the method of Liu et al. [26]. TS patients were recruited from the pediatric department of TCM, Provincial Hospital Affiliated to Shandong University. The presence of antineural antibodies in sera was completed by ELISA kit (R&D, Shanghai, China) in the clinical laboratory at the Provincial Hospital Affiliated to Shandong University. Twenty-four rats with stereotyped behavior were randomly and equally divided into a NDG group and a Hal group, which were treated with IG administration of NDG (370 mg/kg/day) and Hal (1.0 mg/kg/day) for eight weeks, and the medication was started at the end of the 72-hour sera infusion. The rest of the 12 model rats without medication was regarded as the treatment control (TS group), and 12 normal rats were microinfused with normal sera as the model control (CTR group).

Rats in the NDG, Hal, TS, and CTR groups all were anesthetized by intraperitoneal (ip) injection of ketamine (80 mg/kg) and xylazine (10 mg/kg) and placed in a stereotaxic apparatus (Stoelting, USA) with the incisor bar set at 3.5 mm below the interaural line. Then, using an aseptic surgical technique, the skull was exposed and holes were drilled (anterior-posterior 2.0 mm from bregma and medial-lateral ± 4.0 mm), through which a 28-gauge guide cannulae was passed and bilaterally implanted into the striatum (cannulae implant dorsoventral depth 7.0 mm from the skull). An osmotic minipump (Alzet Corp., Palo Alto, CA) was connected to each cannula by a polyethylene tube loaded with 50 *μ*L of undiluted TS or normal sera under sterile conditions. Sera were microinfused at a rate of 0.5 *μ*L/hour for 72 hours.

The sera were isolated to measure the HVA concentration by ELISA at the start (week 0) and the end (week 8) of the study. During the eight-week treatment period, the stereotypic TS behavior was recorded once a week according the methods of Lv et al. [22, 27]. The first record started before the sera infusion (week 0) and then recorded once a week after medication. Finally, all the rats were euthanized under anesthesia, and the striatum was dissected and stored at −°C.

### 2.3. Preparation of NDG

NDG is a preparation used in TCM, which consists of four different plant species, three animal substances, and human placenta, as shown in Table 1. The extraction process was performed according to the procedure of Li et al. [21–27]. The formulation was synthesized with 999 Single Chinese Herbal Extracts and provided by China Resources Sanjiu Medical and Pharmaceutical Co., Ltd. (Shenzhen, China).

### 2.4. Stereotyped Behavior

Rat movements were videoed for 30 minutes once a week after medication, but the first record started before sera infusion (week 0). Several categories of stereotyped behavior were selected from established experimental protocols [22, 29]. Stereotyped behavior included bites (teeth touching the cage, wood chips, and vacuous chewing of other objects except the body), taffy pulling (raising the forepaws to the mouth and face), self-gnawing, licking not associated with grooming, head shaking, paw shaking, rearing, and episodic utterances. Scores ranged from 0 to 5 and were averaged for each item according the method of Lv et al. [22, 27]. In the study, testing for stereotyped behavior was performed by a trained technician, who was blind for enrolled groups.

### 2.5. Striatal DA and Sera HVA Expression Levels

The levels of HVA in sera and of DA in striatal homogenates were measured using a Sandwich enzyme-linked immunosorbent assay (ELISA) according to the manufacturer's instructions (USCN, Wuhan, China). In brief, the striatal tissue was washed twice with ice-cold PBS, and each 100 mg of homogenized striatal tissue was diluted with 1 mL of normal saline (0.9%). Antigen standards and samples were distributed into 96-well plates precoated with the primary antibodies. After adding Biotin Conjugate Reagent and Enzyme Conjugate Reagent into each well, the plates were incubated at 37°C for 60 minutes. Then, the plates were rinsed three times with distilled water. After the chromogenic reaction, absorbance was measured at 450 nm using a microtiter plate reader within 30 minutes.

### 2.6. Immunohistochemical Detection of DAT in the Striatum

Striatal tissue in 4 rats randomly in each group was excised, fixed in formalin overnight, and embedded in paraffin. The fixed striatal tissue was cut into 10 *μ*m slices in the coronal plane. The slices dissolved the paraffin in turpentine and washed three times in PBS (pH 7.4) for 5 minutes each. Then, the slices were incubated with a blocking solution (10% goat serum in PBS) at room temperature for 30 minutes. The endogenous peroxide activity was quenched with a 0.3% hydrogen peroxide solution. Next, the pretreated slices were incubated overnight at 4°C with a primary antibody for rabbit polyclonal DAT (dilution 1 : 600; Chemicon, USA) and with PBS instead of the primary antibody in the null group. The following day, the slices were washed three times in PBS (pH 7.4) for 10 minutes and then incubated with a goat anti-rabbit secondary antibody (dilution 1 : 100; Zhongshan, Beijing, China) for 2 hours at room temperature. Color detection was performed by the addition of 0.05% 3,3′-diaminobenzidine (Sigma, USA) and 0.01% hydrogen peroxide at room temperature. Sections were then lightly counterstained with 0.1% hematoxylin. The OD value of DAT expression in the positive area was determined using Image-Pro Plus 6.0 software (Media Cybernetics, Inc., USA).

### 2.7. Protein Analysis by Western Blotting

The tissue of the rat striatum (approximately 30 mg) was homogenized at 4°C. The tissue was cut, homogenized, and washed in ice-cold PBS twice for 10 minutes at 4°C, and then, the debris was removed by centrifugation for 10 minutes at 20,000 ×g at 4°C. The protein concentration in the supernatant was determined with a BCA-100 Protein Quantitative Analysis Kit (Shenneng, Shanghai, China). Equal amounts of protein (30 *µ*g) were subjected to electrophoresis on 10% SDS-polyacrylamide gels and then transferred to polyvinylidene difluoride membranes by electroblotting. The membranes were first incubated in blocking solution (5% skim milk) for 1 hour at room temperature and then incubated overnight at 4°C with the primary antibodies anti-DAT (1 : 500-dilution, Chemicon, USA). After washing three times with TBST (10 mM Tris-HCl, 0.15 M NaCl, 8 mM sodium azide, 0.05% Tween-20, pH 8.0), the membranes were incubated with horseradish peroxidase-conjugated secondary antibodies (1 : 5,000 dilution, Zhongshan, China) for 1 hour and then washed three times with TBST. Finally, the protein bands were visualized with the ImageQuant LAS 4000 detection system (GEHC, Sweden). As an internal control, glyceraldehyde 3-phosphate dehydrogenase (GAPDH) was detected with anti-GAPDH antibodies. The results were quantified using Image-Pro Plus 6.0 software (Media Cybernetics, Inc., USA).

### 2.8. mRNA Isolation and Quantitative Real-Time PCR Analysis

Total RNA was extracted from the striatal tissue of the rats (approximately 30 mg) with Trizol (Invitrogen, CA) reagent according to the manufacturer's protocol. First-strand cDNA was generated with a commercial Takara RT kit (Takara, Dalian, China) and amplified by real-time PCR with the Quantitest SYBR Green kit (Takara, Dalian, China) and the ABI Prism 7500 real-time PCR instrument and software (Applied Biosystems, CA). The DAT forward primer was 5′-TTGGGTTTGGAGTGCTGATTGC-3′, and the reverse primer was 5′-AGAAGACGACGAAGCCAGAGG-3′ (126 base pairs). All quantifications were performed with rat (GAPDH) as an internal standard. The GAPDH forward primer was 5′-GGTCGGTGTGAACGGATTTGG-3′, and the reverse primer was 5′-TCGCTCCTGGAAGATGGTGATG-3′ (226 base pairs). The reaction of reverse transcription was performed for at 37°C for 15 minutes, 85°C for 5 seconds, and 4°C for 20 minutes. The PCR was performed for 40 cycles at 95°C for 15 seconds, 60°C for 30 seconds, and 72°C for 30 seconds. The relative quantification of mRNA expression was analyzed using the 2-Delta Delta C(T) method [30].

### 2.9. Statistical Analysis

All data are presented as the means ± the standard deviations in the text. Statistical differences among groups were evaluated by one-way ANOVA. Stereotyped behavior score among groups was analyzed by repeated-measures ANOVA. Correlations were analyzed with Pearson correlation coefficients. All analyses were performed with the SPSS statistical software package (Version 18.0, SPSS Inc., Chicago, IL, USA), and *P* < 0.05 was considered statistically significant.

## 3. Results

### 3.1. Stereotyped Behavior

**Figure 1:**
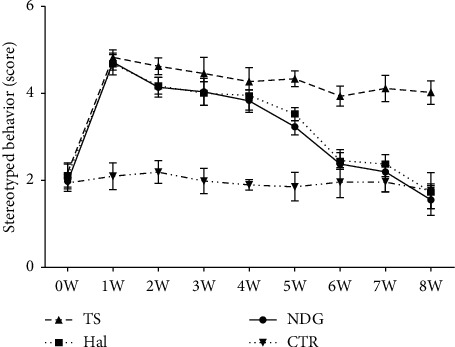
The behavior score was recorded at the start (week 0) and the last day of each week during the medication. Data were presented as mean ± SD (*n* = 12). There was no significant difference among the four groups at week 0. A week later, the scores of stereotyped behavior in both NDG and Hal groups were, respectively, significantly lower than the TS group, and score of the two groups continued to reduce until week 8. Repeated-measures ANOVA revealed that the NDG-treated rats had a lower score compared with nontreated TS rats (<0.05) and no significant differences with Hal group (*P* > 0.05). Score of TS group was significantly higher than the other three groups (NDG, Hal, and CTR) (*P* < 0.01).

Stereotyped behavior of rats was assessed for an eight-week period. The stereotyped behavior scores among groups were analyzed by repeated-measures ANOVA (Figure 1). By the second week following treating with NDG, the stereotyped behavior scores of TS animals were significantly reduced. Moreover, the NDG-treated animals continued to improve until week 8. Statistical result showed that the stereotyped behavior score decreased significantly in the NDG group compared to the TS group (*P* < 0.01). However, no significant differences existed between the NDG and Hal groups from week 0 to week 8 (*P* > 0.05). In addition, significant score was higher in the TS than in the CTR, Hal, and NDG groups (*P* < 0.01).

### 3.2. Striatal DA and Sera HVA Expression Levels

**Figure 2:**
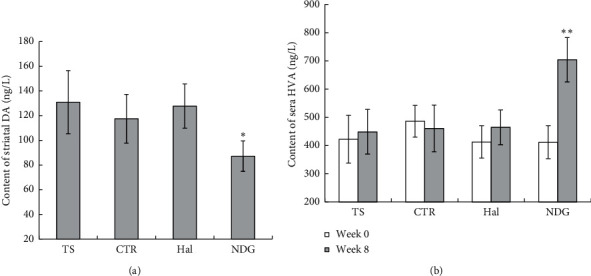
DA expression level in striatal homogenate at week 8. Compared to NDG, the content of DA was significantly higher than that in control, Hal, and CTR (a). Compared to week 0, the content of HVA significantly increased after medication at week 8 in NDG, but no significant difference existed in the Hal, CTR, and TS (b). ^*∗*^*P* < 0.05; ^*∗∗*^*P* < 0.01.

The expression levels of DA and HVA were measured by ELISA. DA levels were found significantly lower in the NDG rat striatum (87.2 ± 12.4 ng/L) related to Hal (127.8 ± 18.0 ng/L) and TS (130.9 ± 25.6 ng/L) groups of rats (*P* < 0.05) (Figure 2(a)).

The concentration of HVA in the sera was measured at week 0 and week 8 (Figure 2(b)). Compared to week 0 (411.5 ± 84.4 ng/L), the expression level of HVA in the sera significantly increased at week 8 (704.4 ± 79.1 ng/L) in the NDG (*P* < 0.01). However, no significant differences existed for sera HVA concentration at week 0 and week 8 in each group of Hal (week 0 : 412.7 ± 56.1 ng/L and week 8 : 464.8 ± 82.7 ng/L), CTR (week 0 : 486.4 ± 57.2 ng/L and week 8 : 460.9 ± 61.6 ng/L), and TS (week 0 : 422.3 ± 58.5 ng/L and week 8 : 448.7 ± 78.6 ng/L).

### 3.3. DAT Protein Expression Levels

**Figure 3:**
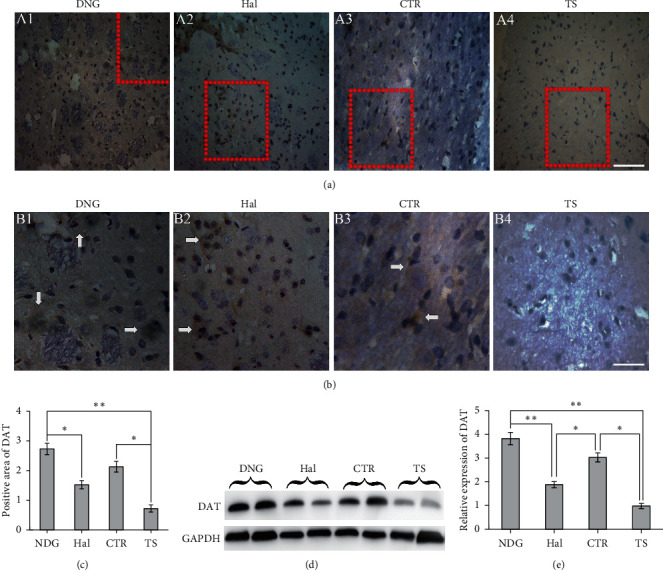
DAT expression in different groups at week 8. Immunochemistry labeling for DAT in different groups was performed at week 8 during medication (n = 5/group). Insets in (A1-A4) are shown at high magnification in (B1-B4); the arrows point to the area with DAT positive expression (A1-B4). Scale bars = 50 *μ*m in (A4) (applies to A1-A4); scale bars = 25 *μ*m in (B4) (applies to B1-B4). Graphical representation shows more positive area of DAT in the NGD-treated group than the Hal and TS group (C) (n = 5). Representative western blots of DAT expression (n = 5/group), and GAPDH was a loading control (D). Semiquantitative measurements were obtained by normalizing GAPDH loading (E). ^*∗∗*^*P* < 0.05^*∗∗*^*P* < 0.01. CTR stands for the CTR group microinfused by normal sera; TS stands for the TS model control without medication; Hal stands for the Haloperidol treatment group; NDG stands for the NDG treatment group.

To determine whether NDG could promote expression of DAT in TS rats, we performed immunohistochemistry and Western blotting for DAT at eight weeks during medication. Positive expression appeared as fine particles in weakly expressing areas and brown stain in strongly expressing areas (mainly localized in the cytoplasm). The OD calculation of DAT expression in the positive area was performed with Image-Pro Plus 6.0 software (Media Cybernetics, Inc., USA) (Figures 3(a)–3(c)). Among the four groups, the OD value in the TS group was the lowest (1.12 ± 0.12) at week 8 (Figure 3(c)). The OD value of positive area was higher in the NDG (2.73 ± 0.30) than in the TS group (*P* < 0.01), but there was no significant difference between the Hal (1.52 ± 0.14) and the TS group (Figure 3(c)). Furthermore, the OD value of DAT positive area in the CTR group (2.23 ± 0.25) was significantly higher than in the TS group (*P* < 0.05). The developed bands for positive DAT expression in Western blots tests were shown in Figure 3(d), and the OD value of developed bands for DAT expression in the positive area was analyzed with the Image-Pro Plus 6.0 software (Media Cybernetics, Inc., USA) shown in Figure 3(e). The OD value of developed bands for DAT positive expression was higher in the NDG group (3.86 ± 0.21) than the Hal group (1.84 ± 0.15) and the TS group (0.98 ± 0.11) (*P* < 0.01). However, there was no difference of OD value of developed bands between the Hal and TS groups (Figure 3(e)).

### 3.4. mRNA Isolation and Quantitative Real-Time PCR Analysis

**Figure 4:**
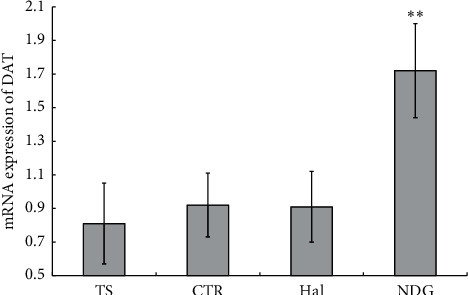
Relative mRNA expression of DAT was analyzed by the 2-Delta Delta C(T) method. Compared to the control group, significantly different from the NDG group (^*∗∗*^*P* < 0.01), but no significant difference from the Hal group and the CTR group.

As shown in Figure 4, the result of real-time PCR was analyzed using the 2-Delta Delta C(T) method [30], and mRNA expression for DAT in the striatum was higher in the NDG group (1.72 ± 0.28) than in the TS group (0.81 ± 0.24) at week 8 (*P* < 0.01). However, there was no significant difference among Hal (0.91 ± 0.21), CTR (0.92 ± 0.19), and TS groups.

### 3.5. Correlation Analyses

**Figure 5:**
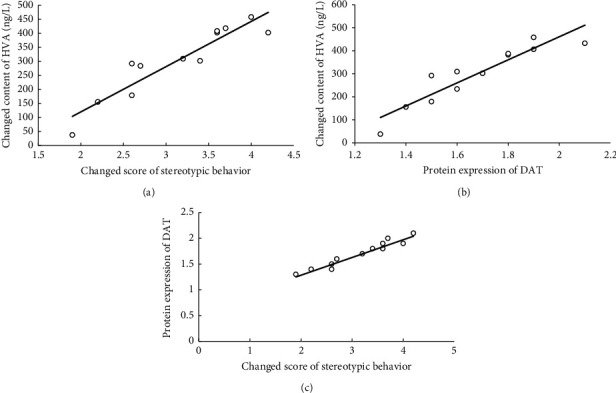
The relationship among the expression of DAT and HVA and score of stereotyped behavior in the NDG group. Scatterplot of correlative analysis between the increased HVA concentration in sera and the DAT protein expression at week 8 showing a positive correlation in the NDG group (r = 0.917, n = 12, *P* < 0.01) (a). Scatterplot of correlative analysis between the improved score of stereotyped behaviors and the DAT protein expression at week 8 showing a positive correlation in the NDG group (r = 0.895, n = 12, *P* < 0.01) (b). Correlative analysis scatterplot between the improved score of stereotyped behavior and the increased HVA concentration in sera showing a positive correlation upon an 8-week period of medication in the NDG group (r = 0.936, n = 12, *P* < 0.01) (c).

As shown in Figures 5(a) and 5(b), correlations of the NDG group were determined with the Pearson correlation coefficients for stereotyped behavior (week 1 score–week 8 score), the concentration (week 8 concentration–week 0 concentration) of sera HVA and striatal DAT protein expression at week 8. The increased HVA sera concentration positively correlated with the decreased score of stereotyped behavior after 8 weeks' medication with NDG (*P* < 0.01). The increased HVA sera concentration also positively correlated as well as higher DAT protein expression at week 8 (*P* < 0.01). Moreover, higher protein DAT expression was also associated with improved score for stereotyped behavior (Figure 5(c)) (*P* < 0.01). However, these correlations were not found in the Hal group.

## 4. Discussion

Accumulating evidence indicates that hyperfunction of the DA system in CSTC circuits plays an important role in TS [16, 29, 31–33]. Additionally, autoantibodies reacting in the brain and immune dysregulation may also accompany the pathophysiology of TS [14–16]. Interestingly, sera enriched with antineural antibodies can induce TS stereotyped behavior in rats [26–28]. In our present study, we also found the similar stereotyped behavior in rats with microinjection into striatum, which might be concomitant with the pathophysiological changes in immune dysregulation in TS.

As a type of transmitting glycoprotein localized exclusively in the presynaptic membrane of DA neurons, DAT is responsible for modulating the concentration of extrasynaptic DA by neuronal reuptake. Therefore, DAT plays an important role in maintaining the phasic tonic balance of DA release [29]. Our previous finding indicated a lower expression of DAT in the TS animals compared to healthy controls [27]. Homovanillic acid (HVA) is the main DA metabolite, and its plasma levels have been widely used to study the function of central DA in psychiatric disorders [34, 35]. Therefore, we deduced and tested whether NDG could regulate DAT expression and mediate the metabolism of DA in the CSTS circuits of TS rats.

Hal, a blocker of DRD_2_, is effective in improving tic symptoms [18]; however, a considerable number of TS children cannot tolerate their adverse reaction [20, 36]. Therefore, these children need an alternative, safe, and effective medication. TCM is widely used for the treatment of diseases, including TS, in China. NDG, a TCM preparation, can reduce stereotyped behavior and regulate metabolic DA in TS animals and ADHD patients [5, 22, 26, 37, 38]. However, its mechanism remains obscure.

In the current study, we evaluated the efficacy of NDG on stereotyped behavior in rats. We compared NDG treatment to Hal because Hal is known to be effective in treating TS symptoms by inhibiting the excitability of cortical motor areas and restraining the activity of DA receptors as a blocker of DRD_2_. However, Hal is not widely used to treat TS children because of its toxic side effects, including sedation, weight gain, extrapyramidal symptoms, and QT interval prolongation evidenced by electrocardiogram [19, 20, 36, 39]. A small number of TS children do not respond to Hal. There was also an inconsistent report with the foregoing illustration that Hal could not improve stereotyped behavior [40], which may have been the result of differences between the sample statistics or interspecies differences between human and rat. Based on our present clinical trials, NDG has a significant effect in TS and ADHD patients without apparent toxic side effects [5, 22–24, 37, 41, 42]. Lv et al. reported that NDG could effectively inhibit the stereotyped behavior in TS rats, and the mechanisms may be related to the improved metabolic disturbance of dopamine by increasing the content of sera HVA, decreasing the content of DA and repressing the expression of DRD_2_ mRNA in the striatum [22]. However, the mechanism by which NDG increases sera HVA and decreases striatal DA concentrations remains unknown. Elucidating the definitive mechanisms of NDG on DAT and DA metabolism may help identify potential targets for the treatment of TS. Furthermore, we induced stereotyped behavior by microinjection into striatum with TS children's sera, which was different from the report of Lv et al. [22].

Proper dopaminergic neurotransmission can mediate many physiologic processes, including addiction, movement, and lactation [27, 43]. In the process of DA metabolism in CSTC circuits, DAT is also an important monoamine neurotransmitter transporter, which is involved in the metabolic pathway of DA [27, 44, 45]. DAT plays a key role in shaping neurotransmission mediated by removing extracellular dopamine and recycling it back into the neuron in the dopaminergic nigrostriatal pathways [44]. After reuptake by DAT, DA is transformed into HVA in the neuron and released into the blood [22, 27]. Thus, according to our data, we presumed that NDG increased HVA content in the sera and decreased the DA content in the striatum of the TS rats, which could be associated with NDG upregulating the protein and mRNA expression of DAT in striatum. Following DAT upregulation, redundant DA was taken up into the neuron, and the content of DA was significantly reduced in the synaptic cleft of neurons in the striatum. Because redundant DA was taken up into the neuron, transformed into HVA, and then released into the blood, this led to increase in HVA in the sera.

Correlation analyses in our studies demonstrated that a positive correlation existed between the improved stereotyped counts and increased HVA concentration in sera and upregulated DAT expression in the striatum after an eight-week treatment course with NDG. The results reveal that NDG could regulate DAT expression and mediate DA metabolism in the striatum and finally improve TS symptoms. Although Hal also improved the score of the stereotyped behavior, it had no effect on sera HVA content or DA concentration and DAT expression in the striatum. More importantly, the correlations between stereotyped behavior score and the concentration of sera HVA or striatal DAT protein expression were not found in the Hal group, which implied that NDG and Hal had different therapeutic mechanisms on TS.

NDG is effective for TS and may have multiple target action for its mechanism as a compound TCM preparation. Additionally, another research by Wang and Li reported that NDG improved stereotyped behavior and its working mechanism involved in regulation of DA metabolism for binding D_2_R decrement and DAT increment by PET, which was conducted by different experimental methods and models [46]. Interestingly, Wang et al. reported that another different TCM formula “Jian-Di-Zhi-Dong Decoction” (JPZDD) could effectively inhibit the abnormal behavior in mice and had similar mechanism for increment of DAT expression in striatum although they chose the different research methods and different models and interventions [47].

In conclusion, NDG attenuates stereotyped behavior, and its mechanism of action might be associated with the upregulation of DAT expression to regulate DA metabolism in the brain, which may also promise implications for drug development in the treatment of TS in the future.

## Figures and Tables

**Table 1:** Composition of Ningdong granule.

Components	Production lot	Part used	Amount used (g)
*Uncaria rhynchophylla* (Miq.) Jacks	201009121	Ramulus	20
*Gastrodia elata* Blume	201004031	Root	6
*Ligusticum chuanxiong* Hort	200912241	Rhizome	6
*Buthus martensii* Karsch	201004161	Dried body	3
*Scolopendra subspinipes mutilans* L. Koch.	201005091	Dried body	Single band
*Haliotis diversicolor* Reeve.	201006171	Shell	20
*Dried human placenta*	201008221	Dried placenta	3
*Glycyrrhiza uralensis* Fisch.	201002291	Rhizome	3

## Data Availability

All data generated during the project will be made freely available via Shenzhen People's Hospital Data Repository. DOIs to these data will be provided (as part of the DataCite programme) and cited in any published article using these data and any other data generated in the project. There are no security, licensing, or ethical issues related to these data.

## References

[1] Cohen S. C., Leckman J. F., Bloch M. H. (2013). Clinical assessment of Tourette syndrome and tic disorders. *Neuroscience & Biobehavioral Reviews*.

[2] Giraldo B. O., David M., Sánchez Y., Miranda J., Sierra J. M., Cornejo J. W. (2013). Prevalence of tics among 6- to 12-year-old schoolchildren in the Itagüi municipality, Colombia, in 2010. *Journal of Child Neurology*.

[3] Singer H. S., Walkup J. T. (1991). Tourette syndrome and other tic disorders diagnosis, pathophysiology, and treatment. *Medicine*.

[4] Leckman J. F. (2002). Tourette’s syndrome. *The Lancet*.

[5] Quezada J., Coffman K. A. (2018). Current approaches and new developments in the pharmacological management of Tourette syndrome. *CNS Drugs*.

[6] Borison R. L., Ang L., Hamilton W. J., Diamond B. I., Davis J. M. (1983). Treatment approaches in gilles de la Tourette syndrome. *Brain Research Bulletin*.

[7] Robertson M. M., Eapen V., Cavanna A. E. (2009). The international prevalence, epidemiology, and clinical phenomenology of Tourette syndrome: a cross-cultural perspective. *Journal of Psychosomatic Research*.

[8] Beste C., Münchau A. (2017). Tics and Tourette syndrome-surplus of actions rather than disorder?. *Movement Disorders*.

[9] Plessen K. J. (2013). Tic disorders and Tourette’s syndrome. *European Child & Adolescent psychiatry*.

[10] Polyanska L., Critchley H. D., Rae C. L. (2017). Centrality of prefrontal and motor preparation cortices to Tourette Syndrome revealed by meta-analysis of task-based neuroimaging studies. *NeuroImage: Clinical*.

[11] Singer H. S., Wendlandt J. T. (2001). Neurochemistry and synaptic neurotransmission in Tourette syndrome. *Advances in Neurology*.

[12] Rampello L., Alvano A., Battaglia G., Bruno V., Raffaele R., Nicoletti F. (2006). Tic disorders: from pathophysiology to treatment. *Journal of Neurology*.

[13] Patel A., Zhu Y., Kuzhikandathil E. V., Banks W. A., Siegel A., Zalcman S. S. (2012). Soluble interleukin-6 receptor induces motor stereotypies and co-localizes with gp130 in regions linked to cortico-striato-thalamo-cortical circuits. *PloS One*.

[14] Murphy T. K., Kurlan R., Leckman J. (2010). The immunobiology of Tourette’s disorder, pediatric autoimmune neuropsychiatric disorders associated withStreptococcus, and related disorders: a way forward. *Journal of Child and Adolescent Psychopharmacology*.

[15] Hoekstra P. J., Anderson G. M., Limburg P. C., Korf J., Kallenberg C. G., Minderaa R. B. (2004). Neurobiology and neuroimmunology of Tourette’s syndrome: an update. *Cellular and Molecular Life Sciences: CMLS*.

[16] Singer H. S., Minzer K. (2003). Neurobiology of Tourette’s syndrome: concepts of neuroanatomic localization and neurochemical abnormalities. *Brain and Development*.

[17] Martino D., Zis P., Buttiglione M. (2015). The role of immune mechanisms in Tourette syndrome. *Brain Research*.

[18] Hayslett R. L., Tizabi Y. (2005). Effects of donepezil, nicotine and haloperidol on the central serotonergic system in mice: implications for Tourette’s syndrome. *Pharmacology Biochemistry and Behavior*.

[19] Sandor P. (2003). Pharmacological management of tics in patients with TS. *Journal of Psychosomatic Research*.

[20] Fachinetto R., Villarinho J. G., Wagner C. (2007). Valeriana officinalis does not alter the orofacial dyskinesia induced by haloperidol in rats: role of dopamine transporter. *Progress in Neuro-Psychopharmacology and Biological Psychiatry*.

[21] Li J. J., Tang H. X., Yin W. J., Li A. Y. (2013). Effect of ningdong granule on stereotyped behaviors in Tourette syndrome model rats of different Chinese medical syndromes. *Zhongguo Zhong Xi Yi Jie He Za Zhi Zhongguo Zhongxiyi Jiehe Zazhi=Chinese Journal of Integrated Traditional and Western Medicine/Zhongguo Zhong Xi Yi Jie He Xue Hui, Zhongguo Zhong Yi Yan Jiu Yuan Zhu Ban, (2013)*.

[22] Lv H., Li A., Ma H., Liu F., Xu H. (2009). Effects of Ningdong granule on the dopamine system of Tourette’s syndrome rat models. *Journal of Ethnopharmacology*.

[23] Zhao L., Li A.-Y., Lv H., Liu F.-Y., Qi F.-H. (2010). Traditional Chinese medicine ningdong granule: the beneficial effects in Tourette’s disorder. *Journal of International Medical Research*.

[24] Zhang F., Li A. (2005). Dual ameliorative effects of Ningdong granule on dopamine in rat models of Tourette’s syndrome. *Scientific Reports*.

[25] Tang H. X., Li A. Y., Li J. J., Hou G. S., Zhang F. (2014). Effect of Ningdong Granule on the levels of IL -12 and TNF-alpha in children patients with Tourette’s syndrome. *Zhongguo Zhong Xi Yi Jie He Za Zhi Zhongguo Zhongxiyi Jiehe Zazhi=Chinese Journal of Integrated Traditional and Western Medicine/Zhongguo Zhong Xi Yi Jie He Xue Hui, Zhongguo Zhong Yi Yan Jiu Yuan Zhu Ban*.

[26] Liu X., Wang Y., Li D., Ju X. (2008). Transplantation of rat neural stem cells reduces stereotypic behaviors in rats after intrastriatal microinfusion of Tourette syndrome sera. *Behavioural Brain Research*.

[27] Jijun L., Zaiwang L., Anyuan L. (2010). Abnormal expression of dopamine and serotonin transporters associated with the pathophysiologic mechanism of Tourette syndrome. *Neurology India*.

[28] Taylor J. R., Morshed S. A., Parveen S. (2002). An animal model of Tourette’s syndrome. *American Journal of Psychiatry*.

[29] Wong D. F., Brašić J. R., Singer H. S. (2008). Mechanisms of dopaminergic and serotonergic neurotransmission in Tourette syndrome: clues from an in vivo neurochemistry study with PET. *Neuropsychopharmacology*.

[30] Livak K. J., Schmittgen T. D. (2001). Analysis of relative gene expression data using real-time quantitative PCR and the 2−ΔΔCT method. *Methods*.

[31] Hwang W. J., Yao W. J., Fu Y. K., Yang A. S. (2001). [99mTc]TRODAT-1/[123I]IBZM SPECT studies of the dopaminergic system in Tourette syndrome. *Psychiatry Research*.

[32] Pittenger C. (2017). Histidine decarboxylase knockout mice as a model of the pathophysiology of tourette syndrome and related conditions. *Handbook of Experimental Pharmacology*.

[33] Rapanelli M., Frick L. R., Pogorelov V. (2014). Dysregulated intracellular signaling in the striatum in a pathophysiologically grounded model of Tourette syndrome. *European Neuropsychopharmacology*.

[34] Dhir A., Kulkarni S. K. (2007). Involvement of dopamine (DA)/serotonin (5-HT)/sigma (*σ*) receptor modulation in mediating the antidepressant action of ropinirole hydrochloride, a D_2_/D_3_ dopamine receptor agonist. *Brain Research Bulletin*.

[35] Coccaro E. F., Hirsch S. L., Stein M. A. (2007). Plasma homovanillic acid correlates inversely with history of learning problems in healthy volunteer and personality disordered subjects. *Psychiatry Research*.

[36] Oulis P., Karapoulios E., Masdrakis V. G. (2008). Levetiracetam in the treatment of antipsychotics-resistant Tourette syndrome. *The World Journal of Biological Psychiatry*.

[37] Wang S., Qi F., Li J., Zhao L., Li A. (2012). Effects of Chinese herbal medicine Ningdong granule on regulating dopamine (DA)/serotonin (5-TH) and gamma-amino butyric acid (GABA) in patients with Tourette syndrome. *Bioscience Trends*.

[38] Lv H., Lin H.-Y., Zhao H., Li A.-Y., Lin Y., Yao B. (2012). Effects of ningdong granule on DA, DRD_2_, and HVA in a rat model of Tourette’s syndrome. *Journal of Traditional Chinese Medicine*.

[39] Yoo H. K., Kim J. Y., Kim C. Y. (2006). A pilot study of aripiprazole in children and adolescents with Tourette’s disorder. *Journal of Child and Adolescent Psychopharmacology*.

[40] Rajapakse T., Pringsheim T. (2010). Pharmacotherapeutics of Tourette syndrome and stereotypies in autism. *Seminars in Pediatric Neurology*.

[41] Li J. J., Tang H. X., Yin W. J., Li A. Y. (2013). Effect of ningdong granule on stereotyped behaviors in Tourette syndrome model rats of different Chinese medical syndromes. *Zhongguo Zhong Xi Yi Jie He Za Zhi Zhongguo Zhongxiyi Jiehe Zazhi=Chinese Journal of Integrated Traditional and Western Medicine*.

[42] Li A.-Y., Cong S., Lu H., Li J.-J., Zhao L. (2009). Clinical observation on treatment of Tourette syndrome by integrative medicine. *Chinese Journal of Integrative Medicine*.

[43] Kahlig K. M., Galli A. (2003). Regulation of dopamine transporter function and plasma membrane expression by dopamine, amphetamine, and cocaine. *European Journal of Pharmacology*.

[44] Gulley J. M., Zahniser N. R. (2003). Rapid regulation of dopamine transporter function by substrates, blockers and presynaptic receptor ligands. *European Journal of Pharmacology*.

[45] Pan X., Guo X., Xiong F., Cheng G., Lu Q., Yan H. (2015). Acrylamide increases dopamine levels by affecting dopamine transport and metabolism related genes in the striatal dopaminergic system. *Toxicology Letters*.

[46] Wang Y., Li A. (2019). Regulatory effects of Ningdong granule on dopaminergic and serotonergic neurotransmission in a rat model of Tourette syndrome assessed by PE. *Molecular Medicine Reports*.

[47] Wang D. H., Li W., Liu X. F., Zhang J. M., Wang S. M. (2013). Chinese medicine formula “jian-pi-zhi-dong decoction” attenuates tourette syndrome via downregulating the expression of dopamine transporter in mice. *Evidence-Based Complementary and Alternative Medicine*.

